# Favorable pharmacokinetic and tolerability profiles make carprofen an attractive analgesic for subcutaneous injection and oral self-administration in rats

**DOI:** 10.1038/s41598-025-93336-3

**Published:** 2025-03-15

**Authors:** Aylina Glasenapp, Jens P. Bankstahl, Heike Bähre, Andrey Kozlov, Silke Glage, Marion Bankstahl

**Affiliations:** 1 Institute for Laboratory Animal Science and Central Animal Facility, Hannover Medical School, Hannover, Germany; 2 Department of Nuclear Medicine, Hannover Medical School, Hannover, Germany; 3 Research Core Unit Metabolomics, Hannover Medical School, Hannover, Germany; 4 Ludwig Boltzmann Institute for Traumatology, The Research Center in Cooperation with AUVA. Austrian Cluster for Tissue Regeneration, Vienna, Austria; 5 Department of Biological Sciences and Pathobiology, Institute of Pharmacology and Toxicology, University of Veterinary Medicine Vienna, Vienna, Austria

**Keywords:** Pharmacokinetics, Analgesia, Carprofen, Rats, Drinking water, Tolerability, Pain, Preclinical research, Adverse effects, Behavioural methods, Drug delivery, Drug safety, Pharmacology

## Abstract

As basis for evidence-based analgesia refinement, species-specific pharmacokinetic and tolerability profiles of carprofen were determined in rats for least aversive administration routes and prolonged treatment. Further, potential influence on behavioral pain indicators was evaluated. LC-MS/MS determined plasma concentrations in Sprague-Dawley rats (*n* = 21/sex) after subcutaneous (s.c.) injection (5 mg/kg) and during a 5-day treatment via the drinking water (d.w., 10 mg/kg/24 h). Irwin test parameters, clinical scoring, body weight, body temperature, fluid and food intake, grimace scale, burrowing, nesting, hematology, and histopathology were investigated. Plasma concentrations early after injection were higher in females, reached a maximum (C_max_) of 39.16 ± 7.38 µg/ml at 3 h after injection and remained above an estimated in-vitro-derived therapeutic threshold (24.3 µg/ml) for at least 6 h with a T_1/2_ of 7.06 h. Carprofen-medicated d.w. was readily consumed, with constant target dose intake over the 5-day treatment period reaching a C_max_ of 38.68 ± 8.67 µg/ml at 24 h. Tolerability and behavioral parameters revealed only minor changes, such as transient sedation (s.c.) and decreased body temperature (females). Gastrointestinal adverse effects were not detected. Carprofen’s pharmacokinetic profile allows for a practicable s.c. injection interval. Acceptance and tolerability during prolonged oral treatment with the assessed dose of 10 mg/kg/24 h makes its non-invasive administration promising for analgesia refinement in rats.

**Supplementary Information:**

The online version contains supplementary material available at 10.1038/s41598-025-93336-3.

## Introduction

Appropriate analgesia for painful interventions is an important goal for animal welfare and contributes to refinement in animal experiments according to the 3R principles^1^. Moreover, adequate usage can avoid negative effects of pain on experiments and therefore prevent falsified research results^2^. Although awareness for analgesic treatment of laboratory animals emerged, especially rodents might still be undertreated^3–5^ as relevant data on standard analgesics are not available for these species. To derive evidence-based recommendations for analgesic pain management and refinement, it is of high importance to determine pharmacokinetic (PK) and tolerability profiles of commonly used analgesics also in rodents.

Carprofen (CAR) is a nonsteroidal anti-inflammatory drug (NSAID) and widely used both for the management of chronic pain as well as acute soft tissue injury in animals and humans^6^. The analgesic effect is mainly exerted by local inhibition of cyclooxygenase (COX) enzymes resulting in decreased pro-inflammatory mediators. While intravenous injection is the most precise and reliable administration route, without the drawback of reduced bioavailability, it is not very practical for routine pain treatment. Therefore, subcutaneous (s.c.) administration is the most applied route and recommended doses for CAR are currently ranging between 2 and 5 mg/kg every 12–24 h^6,7^. Despite its widespread use, related plasma concentrations and derived PK parameters have not been determined in rats. For non-invasive administration, oral self-intake from the drinking water (d.w.) is highly attractive, as the application is stress-free for the animals and convenient to integrate into daily routine. CAR is a promising candidate for this administration route as it is stable in d.w. for at least 7 days^8^and there is no indication for aversion of rodents to CAR-medicated water^9–11^. However, in the current literature, data and dose recommendations for this administration route in rats are not yet available. Likewise, data on potential side effects in rats are rare. Although NSAIDs such as CAR are generally associated with an increased risk of gastrointestinal ulceration^12^, species-specific structured analysis for rats still remains to be undertaken. Just as little is known on CAR’s potential impact on the outcome of commonly applied behavioral surrogate markers for pain assessment, such as burrowing or nesting, which could lead to a certain distortion in the interpretation of these parameters. As species differences are crucial, it is good pharmacological practice to determine PK and tolerability profiles in the target species.

Consequently, the first objective of this study was to define PK profiles of CAR both after s.c. injection and during voluntary intake from the d.w. in rats as the target species. Further, this study was thought to assess the feasibility and tolerability of prolonged non-invasive CAR administration via the d.w. Moreover, we aimed to assess its potential impact on behavioral pain parameters.

## Results

### Plasma analysis after s.c. injection shows a long plasma half-life, and CAR intake from d.w. results in stable plasma levels

**Fig. 1:**
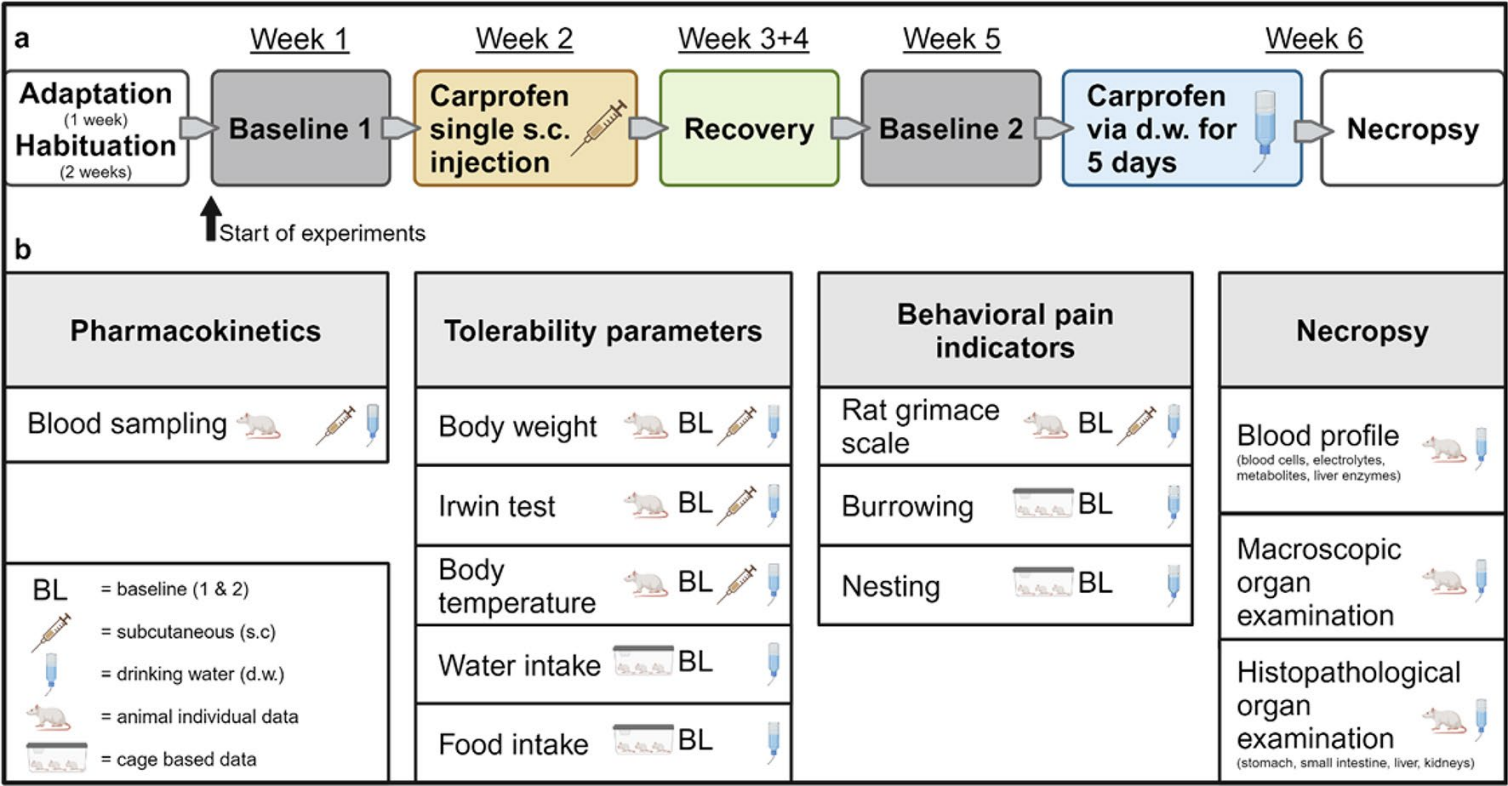
Experimental design. (**a**) Timeline of the complete experimental setup. (**b**) Overview of all interventions and parameters. Created with BioRender.com. BL, baseline; d.w., drinking water.

**Fig. 2:**
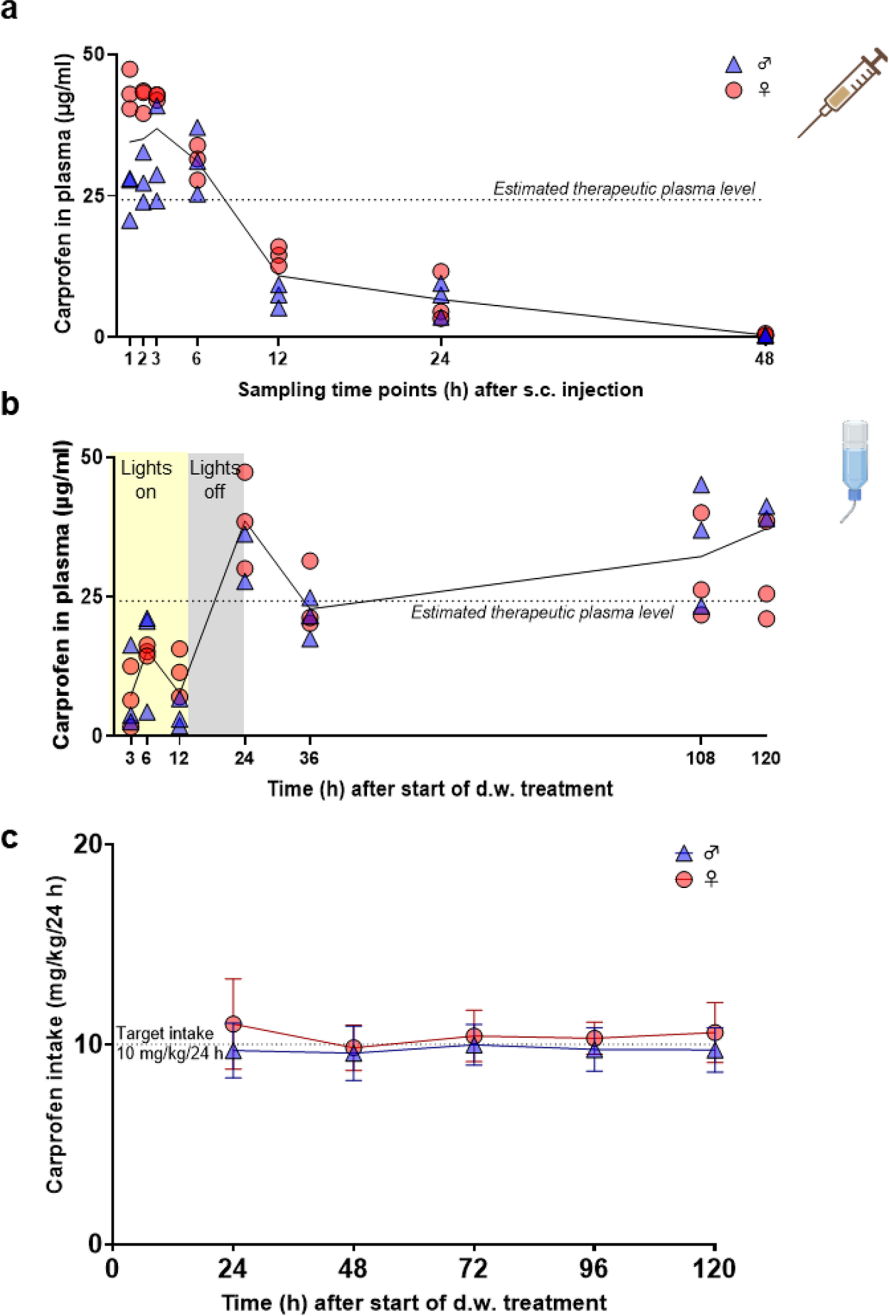
Long plasma elimination half-life following single s.c. injection of 5 mg/kg CAR and stable CAR plasma levels during oral self-administration of 10 mg/kg/24 h. (**a**) Plasma concentration-time curve after s.c. injection is presented. Individual data points (*n* = 6, 3 male and 3 female rats per time point) and mean of all animals per time point (black line) are presented. Estimated minimal therapeutic plasma level (dotted line) is displayed according to reference (13)). (**b**) Plasma concentration-time curve during oral intake of CAR (intended dose: 10 mg/kg/24 h) via d.w. over 5 consecutive days. Individual data points (*n* = 6, 3 male and 3 female rats per time point) and mean of all animals per time point (black line) are presented. Estimated minimal therapeutic plasma level (dotted line) is displayed according to reference (13). (**c**) CAR dose intake from d.w. (mg/kg) per individual rat is displayed for 24 h intervals after treatment start and remains in the area of the target dose intake of 10 mg/kg/24 h (dotted line). Dose intake was calculated from fluid consumption/24 h and concentration of CAR-medicated d.w. (*n* = 7 cages per sex, 3 rats per cage).

An overview of the experimental setup is provided in Fig. 1. To generate PK profiles of CAR in rats, tail vein blood was collected at 1, 2, 3, 6, 12, 24, and 48 h after s.c. injection and at 3, 6, 12, 24, 36, 108, and 120 h after start of oral self-administration via d.w. Single s.c. injection of 5 mg/kg CAR resulted in maximum plasma concentration (C_max_) of 39.16 ± 7.38 µg/ml after 3 h and an elimination half-life of 7.06 h (Fig. 2a). PK parameters are provided in Table 1. Plasma concentrations in most individuals were well above an assumed therapeutically useful level of 24.3 µg/ml, representing the in vitro canine whole blood assay IC_80_value for COX-2 inhibition^13^, for at least 6 h after injection. During the first 3 h after s.c. injection, plasma concentrations in male rats were mostly below those of female individuals (1 h *p* < 0.0001; 2 h *p* = 0.0008; 3 h *p* = 0.0095). In addition, the sampling time points 1, 2, 3, and 12 h after injection showed a negative correlation between bodyweight and CAR plasma concentration, as the heavier males (see also Materials and Methods for average body weight) had lower CAR plasma concentrations than the lighter females (Supplementary Fig. S1). Oral self-administration of CAR (10 mg/kg/24 h) started in the morning of the light phase (Monday morning 7–8 am) and led to plasma levels around or above the estimated therapeutic threshold from 24 h after treatment start until the end of this experiment (at 120 h, Fig. 2b). At 24 h, a C_max_ of 38.68 ± 8.67 µg/ml was observed. The consumed amount of CAR-medicated water was comparable to that of non-medicated d.w. and resulted in uptake of the target dose (male rats: 9.8 ± 1.1 mg/kg/24 h, female rats: 10.4 ± 0.9 mg/kg/24 h) (Fig. 2c, Supplementary Fig. S2a). Since the rats consumed around 80% of total CAR during the dark phase due to circadian rhythm (Supplementary Fig. S2b), it is important to note that plasma levels remained still above or close to the assumed therapeutic threshold at the end of the light phases (36 and 120 h) during the five-day treatment period.

**Table 1:** Noncompartmental analysis of PK parameters after s.c. injection of 5 mg/kg CAR in rats (21 male, 21 female).

Parameter	Unit	Mean	SD
C_max_	µg⋅mL⁻¹	39.16	7.38
T_max_	h	1.67	0.82
T_1/2_	h	7.06	0.53
AUC 0→∞	h⋅µg⋅mL⁻¹	510.12	108
AUMC 0→∞	h²⋅µg⋅mL⁻¹	5693.02	1634.16
MRT 0→∞	h	11.06	1.29
Vd/F	mL⋅kg⁻¹	104.31	26.83
CL/F	mL⋅h⁻¹⋅kg⁻¹	10.2	2.31

C_max_, maximum plasma concentration; T_max_, time of maximum plasma concentration; T_1/2_, plasma elimination half-life; AUC 0→∞ , area under the curve from time 0 extrapolated to infinity; AUMC 0→∞ , area under the first moment curve from time 0 extrapolated to infinity; MRT 0→∞, mean residence time from time 0 extrapolated to infinity; Vd/F , volume of distribution; CL/F, clearance.

### Very good tolerability of CAR after s.c. injection and during prolonged self-administration by d.w

**Fig. 3:**
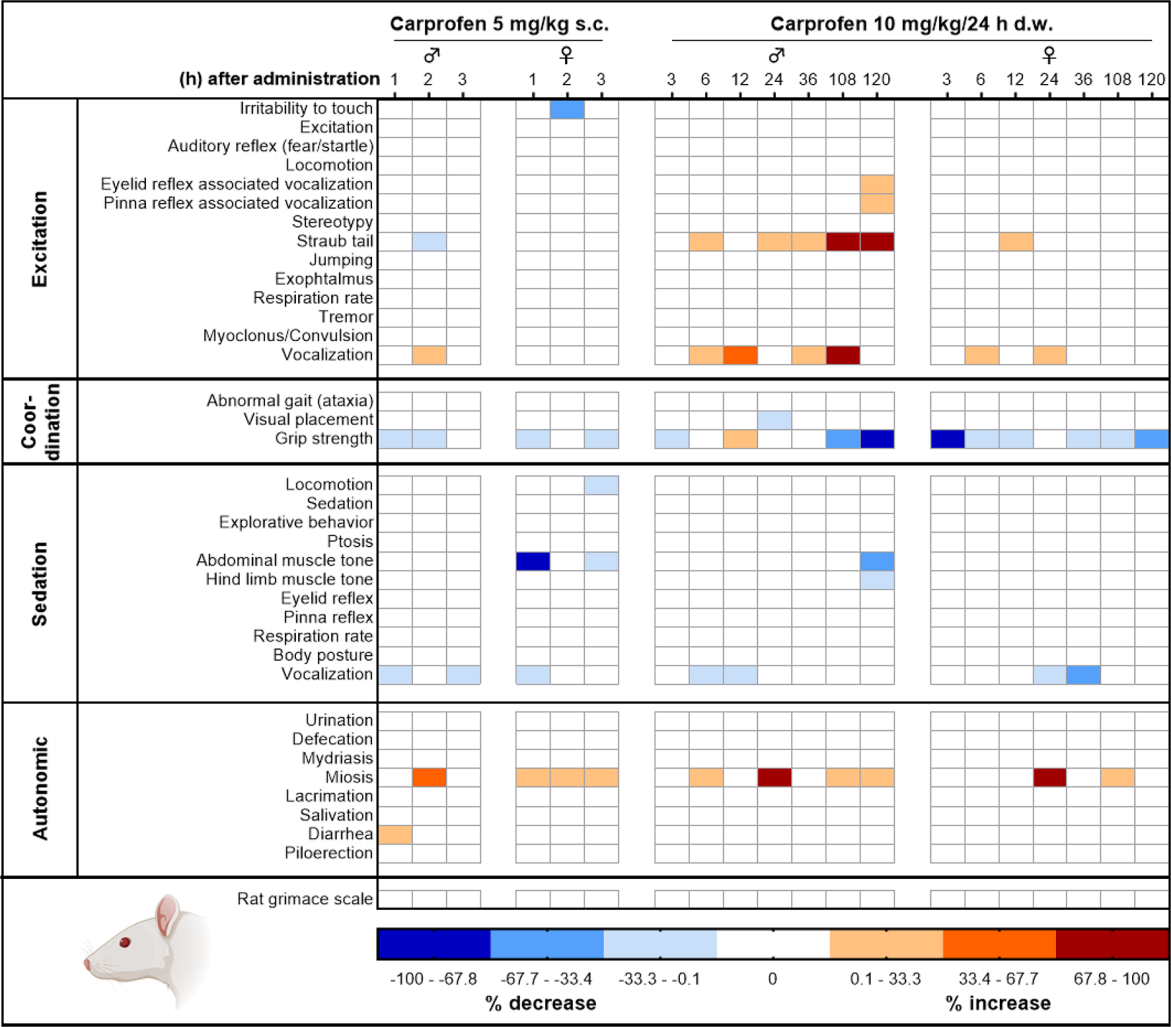
Assessment of CAR tolerability using a modified Irwin test. Effects of CAR treatment on behavioral and physiological parameters are displayed as heat map. Per time point and sex, 3 rats were investigated. Increase or decrease is presented as % change of total number of animals per sex and time point.

**Fig. 4:**
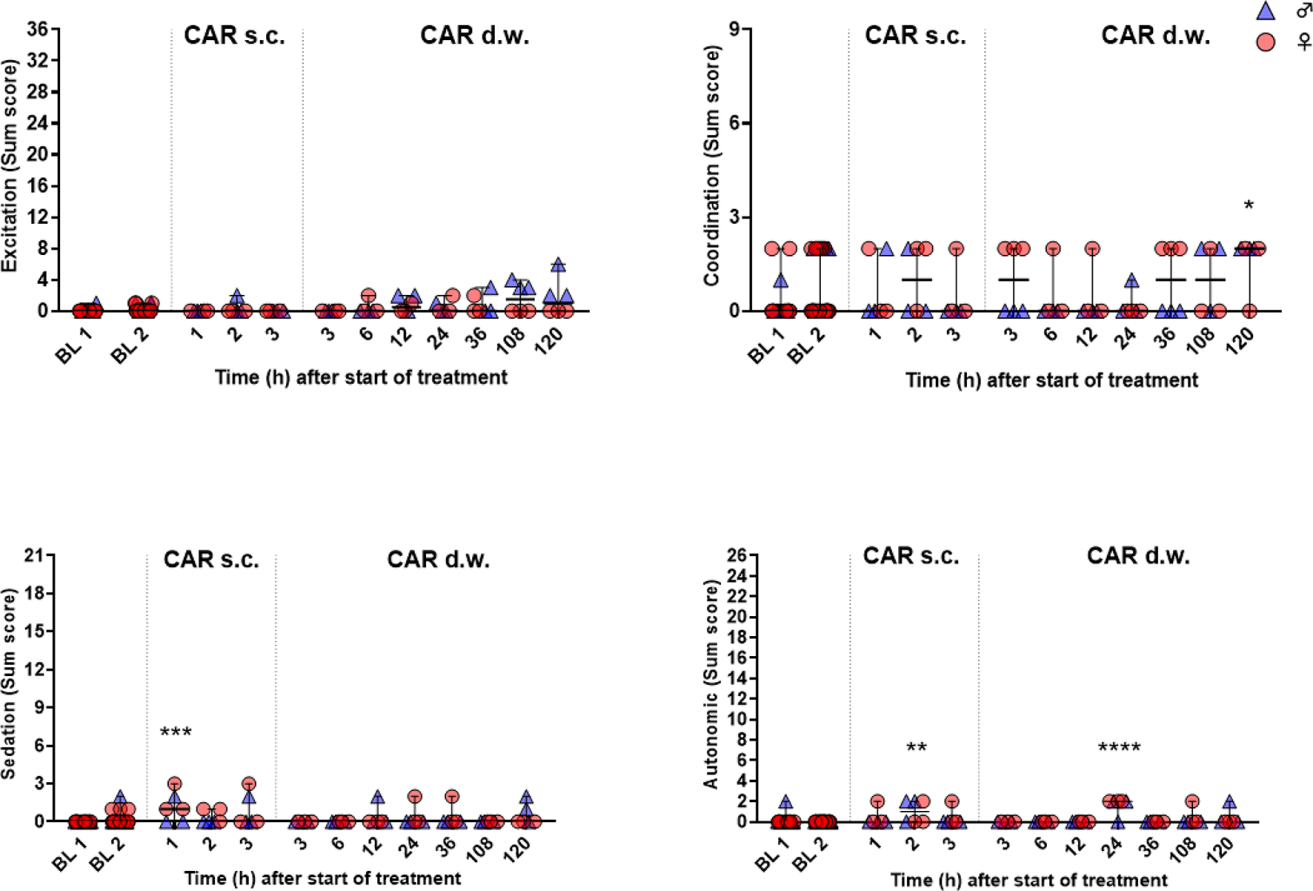
Sum scores of Irwin test categories demonstrate only minor influence of CAR. Sum scores were analyzed in categories excitation, coordination, sedation, and autonomous system. Per time point and sex, 3 rats were investigated. Kruskal-Wallis test followed by Dunn’s multiple comparisons test show slightly but significantly higher sum score values compared to individual baseline at single time points (*p < 0.05; **p < 0.01; ***p < 0.001; ****p < 0.0001).

Tolerability and potential side effects were evaluated by a modified Irwin test protocol^14^ including read-out parameters of different categories, viz. excitation, coordination, sedation, and autonomous system, as well as the rat grimace scale, being displayed in a heat map (Fig. 3). Altogether, only few parameters of the battery were affected. After s.c. injection, both sexes showed a slight decrease in grip strength and vocalization and presented with miosis, whereas female rats also showed reduced abdominal muscle tone. During prolonged treatment via d.w., especially male rats showed signs of excitation, represented by increased handling-associated vocalization and by increased tail elevation (Straub-like). In addition, i.e. independent of the Irwin test assessment, Straub-like tails were observed in 9/21 male rats at the final time point (120 h) in the home cage. On the other hand, handling-induced vocalization was also reduced in some individuals. Further, grip strength was reduced in individuals of both sexes throughout the prolonged treatment. For the categories excitation, coordination, sedation, and autonomous system, a respective sum score for each individual animal and time point is presented in Fig. 4. After s.c. injection, transiently increased sum score values for sedation and autonomous system were observed (sedation: 1 h, *p* = 0.0002; autonomous system: 2 h, *p* = 0.0054). During d.w. treatment, significant changes compared to BL occurred at 24 h for autonomous system (*p* < 0.0001) and at 120 h for coordination (*p* = 0.0146). Live scoring of the rat grimace scale was performed as part of the Irwin test procedure in the home cage and did not reveal increased score values at any time point.

Besides that and without having it assessed in a structured way, increased defecation was observed during burrowing procedure in some individuals at d2 (females 2/21), d3 (males 1/21; females 2/21) and d4 (males 2/21; females 3/21) after treatment start. Very soft feces in the nesting area of the cage was observed at 120 h (males, 2/7 cages).

**Fig. 5:**
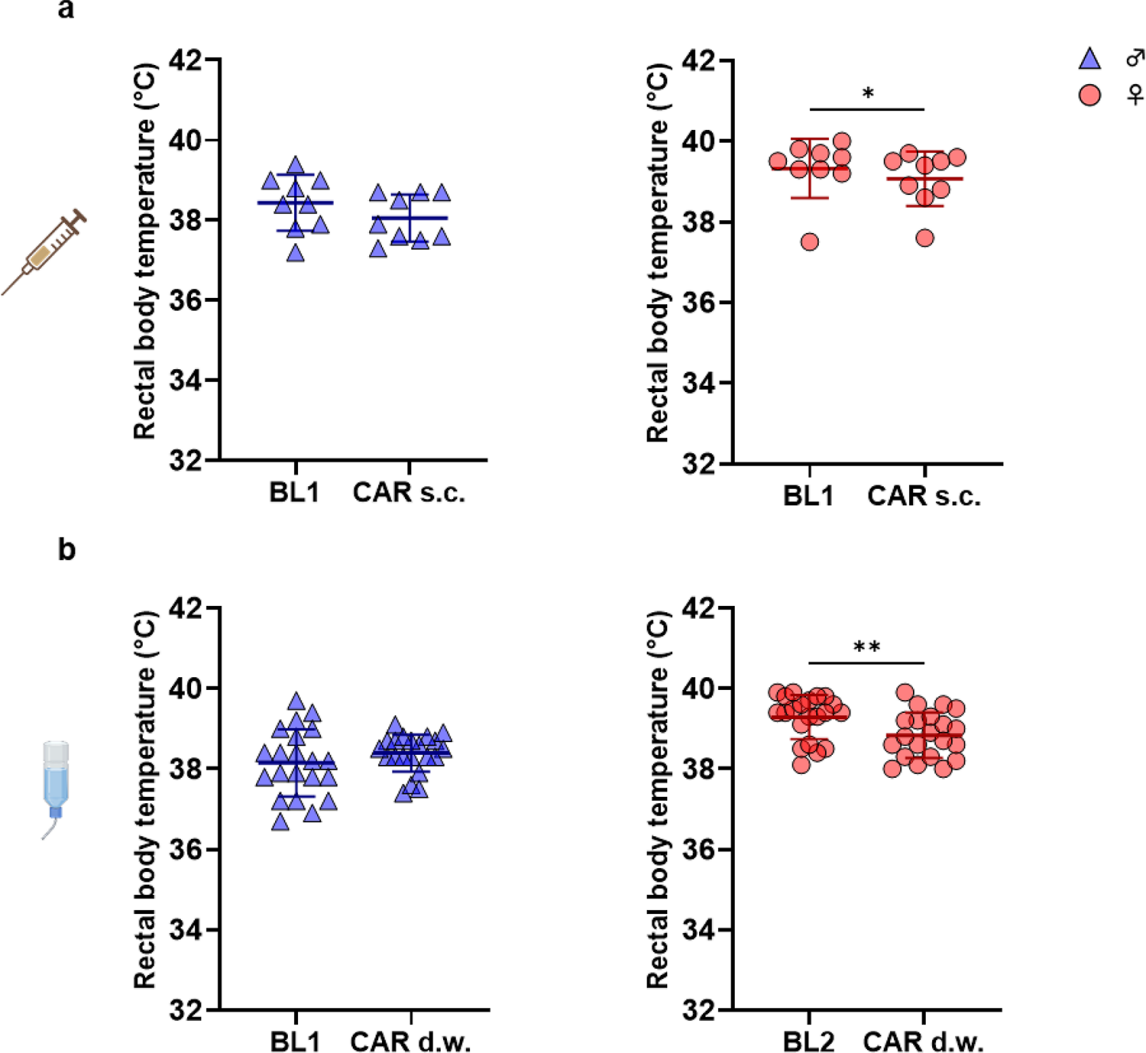
Decreased body temperature in CAR-treated female rats. Rectal body temperature from measurements after (**a**) s.c. CAR treatment and (**b**) during d.w. intake are shown for male (left) and female (right) rats. Wilcoxon matched-pairs signed rank test was used for analysis of paired data per sex after s.c. injection (*n* = 18; 9 male, 9 female rats) and during treatment via d.w. (*n* = 42; 21 male, 21 female rats) and showed decreased body temperature in female rats indicated by asterisk (*p* = 0.0430 and *p* = 0.0047, respectively).

Overall reduced body temperature was found in female but not in male rats after both s.c. injection (BL1 39.32 ± 0.73 °C vs. CAR s.c. 39.07 ± 0.67 °C, *p* = 0.0430) (Fig. 5a) and treatment via d.w. (BL2 39.29 ± 0.55 vs. CAR s.c. 38.83 ± 0.57 °C, *p* = 0.0047) (Fig. 5b). For female rats, no significant difference between baseline data was determined (BL1 39.32 ± 0.73 °C vs. BL2 39.29 ± 0.55 °C, *p* = 0.3594). For male rats, data from BL2 are lacking due to a spontaneous failure of the measurement probe (see Material and Methods).

Water and food consumption as assessed by gravimetric measurement during CAR treatment via the d.w. was stable compared to BL2 measures (Supplementary Fig. S3). Body weight (Supplementary Fig. S4) remained steady and no alterations of clinical score values were observed due to CAR treatment (Supplementary Fig. S5).

### Prolonged oral CAR administration does not impair burrowing and nest building performance

**Fig. 6:**
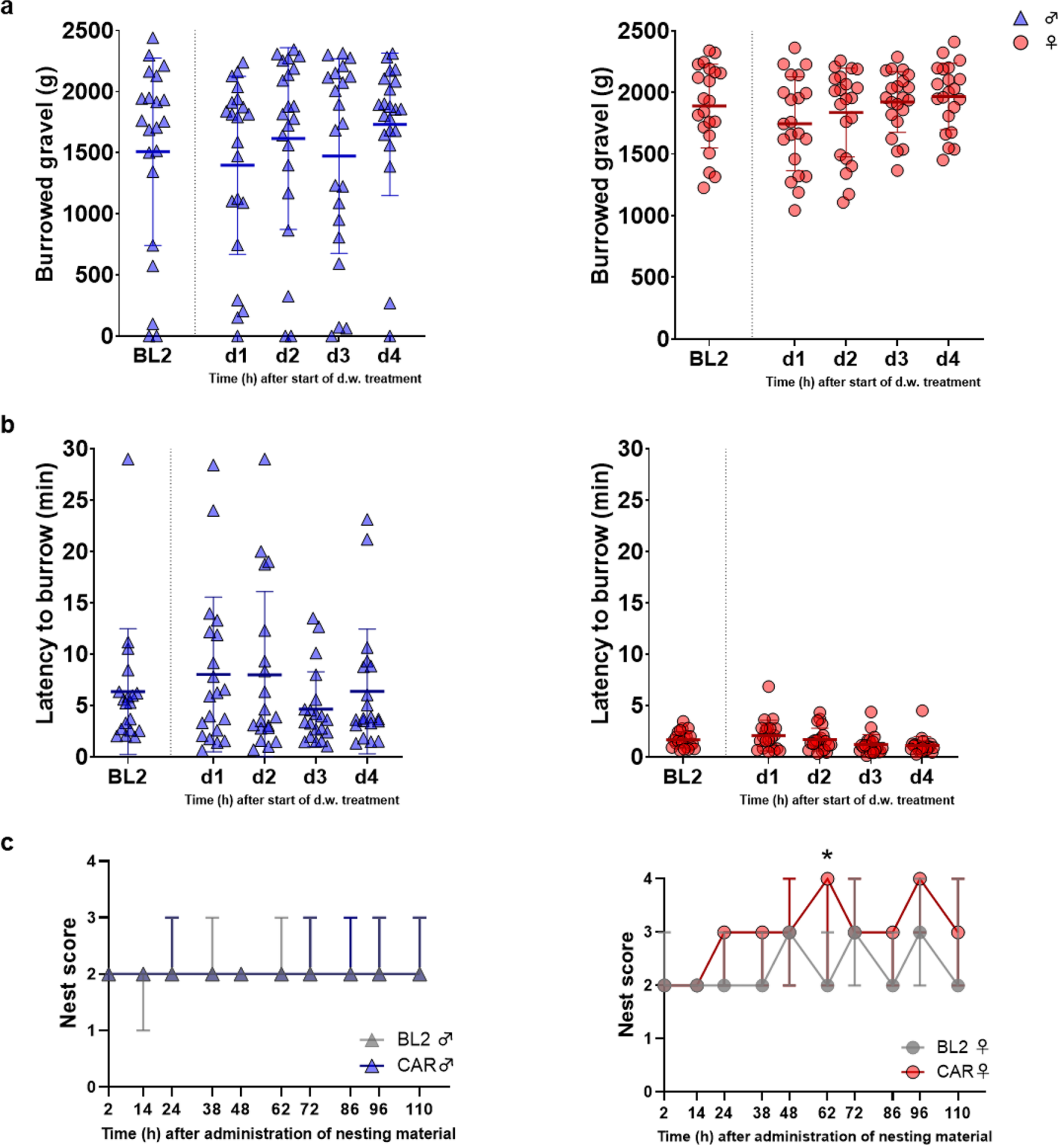
Burrowing and nesting behavior are predominantly not changed by prolonged CAR treatment. (**a**) No changes in amount of burrowed gravel (g) after 30 min of burrowing in male (left) and female (right) rats were observed vs. BL2 (*n* = 21 rats per sex). (**b**) Latency (minutes) to start burrowing behavior for male (left) and female (right) rats is shown (*n* = 21 rats per sex). Data are presented as mean ± SD. One-way ANOVA and post-hoc analysis by Dunnett’s multiple comparisons did not reveal statistical significant differences for burrowing activity after treatment start vs. BL2. (**c**) Nest score values for male (left) and female (right) rats during CAR treatment via d.w compared to BL2 are shown. Fresh nesting material was provided 10 h after start of d.w. treatment. Data are presented as median and range (*n* = 7 cages per sex, 3 rats/cage). Two-way ANOVA followed by Šídák’s multiple comparisons test was performed to test for differences.

To assess the potential influence of prolonged CAR treatment via the d.w. on burrowing behavior, the amount of the originally offered 2500 g gravel burrowed within 30 min and the latency time to start of burrowing was measured. At baseline conditions, female rats burrowed more gravel compared to male rats (BL2; male 1509 ± 767 g vs. female 1890 ± 339 g, *p* = 0.0434), and latency time to start burrowing was significantly shorter in females compared to males (BL 2; male 6.34 ± 6.13 min vs. female 1.67 ± 0.80 min, *p* = 0.0013). Burrowing performance was assessed every day during CAR d.w. treatment. Both the amount of burrowed gravel (Fig. 6a) and the latency to burrow (Fig. 6b) remained unaffected in both sexes.

Nest building performance is presented in Fig. 6c. Nests built by female rats achieved mostly higher score values under CAR d.w. treatment than at baseline conditions, with statistically significant impact at 62 h (*p* = 0.0107) after provision of fresh nesting material.

### Oral CAR treatment did not induce pathohistological alterations or liver enzymes, but slightly altered single blood profile parameters

A standard blood profile after cardiac puncture was measured after 120 h of continuous CAR d.w. treatment and compared to healthy control animals. Blood count of white blood cells (WBC), red blood cells (RBC), hemoglobin (HGB), hematocrit (HCT), platelets (PLT), mean corpuscular volume (MCV), mean corpuscular hemoglobin (MCH), and mean corpuscular hemoglobin concentration (MCH) is provided in Table 2. Unpaired two-tailed Student’s t-test revealed 24% higher PLT cell count (10^3^/mm^3^) (control vs. CAR, *p* = 0.0195), 6% higher MCH (pg) (control vs. CAR, *p* < 0.0001), and 7% higher MCHC (g/dl) values (control vs. CAR, *p* < 0.0001). Analysis of electrolytes and metabolites did not indicate deviations between control and CAR-treated animals. Also, determination of the liver enzymes aspartate transaminase (AST) and alanine transaminase (ALT) did not reveal significant differences between control and CAR-treated rats.

**Table 2:** Blood count, electrolyte, metabolite and enzyme analysis of naïve control rats (7 male, 6 female) and CAR-treated rats (21 male, 21 female) performed after final cardiac puncture.

Parameter	Control (*n* = 13)	CAR (*n* = 42)	Analyzer
WBC (10^3^/mm^3^)	6.81 ± 1.98	6.68 ± 1.65	a
RBC (10^6^/mm^3^)	8.50 ± 1.11	8.30 ± 0.83	a
HGB (g/dl)	14.59 ± 2.26	15.11 ± 1.36	a
	16.18 ± 1.21	15.38 ± 1.25	b
HCT (%)	47.02 ± 6.36	45.86 ± 4.58	a
	49.48 ± 3.67	46.28 ± 3.02	b
PLT (10^3^/mm^3^)	637.4 ± 223.5	789.0 ± 149.7 *	a
MCV (*f*m^3^)	55.38 ± 1.50	55.02 ± 1.57	a
MCH (pg)	17.14 ± 0.88	18.25 ± 0.63 ****	a
MCHC (g/dl)	30.98 ± 1.52	33.04 ± 1.17 ****	a
cK^+^ (mmol/l)	6.37 ± 0.76	6.72 ± 0.79	b
cNa^+^ (mmol/l)	148.9 ± 0.98	147.7 ± 3.79	b
cCa^2+^ (mmol/l)	1.71 ± 0.09	1.76 ± 0.076	b
cCl^-^ (mmol/l)	105.8 ± 1.42	108.3 ± 4.81	b
cGlu (mmol/l)	12.95 ± 8.53	16.54 ± 8.65	b
cLac (mmol/l)	5.35 ± 2.32	6.94 ± 2.13	b
AST (U/L)	171.0 ± 40.57	156.7 ± 58.90	c
ALT (U/L)	38.91 ± 10.55	44.48 ± 17.41	c

WBC, white blood cells; RBC, red blood cells; HGB, hemoglobin; HCT, hematocrit; PLT (platelets); MCV, mean corpuscular volume; MCH, mean corpuscular hemoglobin; MCHC, mean corpuscular hemoglobin concentration; AST, aspartate transaminase; ALT, alanine transaminase. Values presented as mean ± SD; * *p* < 0.05, ** *p* < 0.01, *** *p* < 0.001, **** *p* < 0.0001 (unpaired two-tailed Student’s t-test, control *versus* CAR-treated rats). a, scil Vet abc, scil animal care company GmbH, Germany; b, ABL815 Flex blood gas analyzer, Radiometer, Denmark; Cobas c111, Roche Diagnostics, Vienna, Austria.

**Fig. 7:**
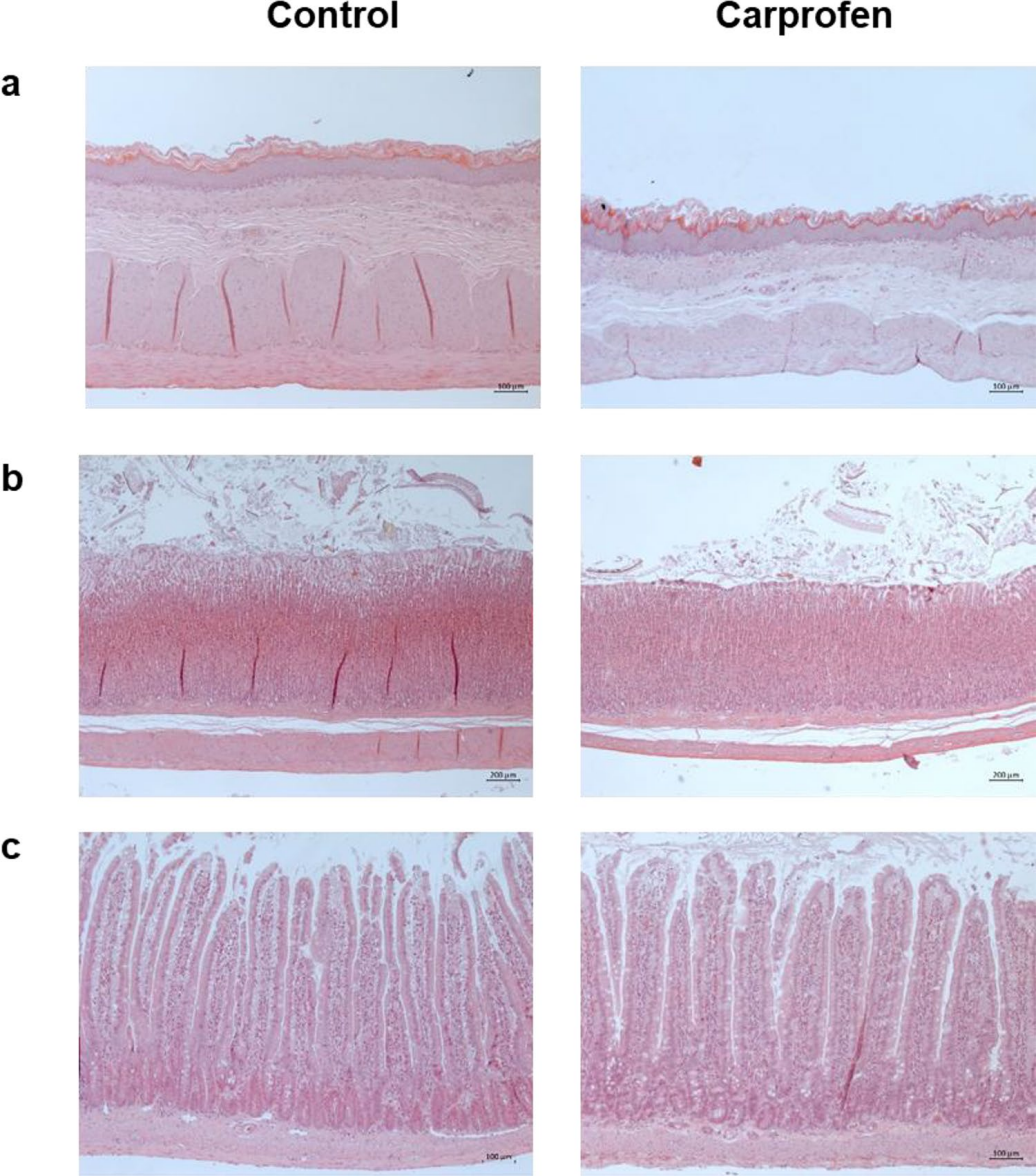
No histopathological alterations after CAR treatment. Representative images of hematoxylin-eosin stained sections of (**a**) forestomach (**b**) glandular stomach and (**c**) proximal part of jejunum of a control animal (left) and a rat after 5 days of CAR treatment (right). No signs of inflammation or ulceration were detected.

To assess potential side effects of continuous CAR treatment for 5 days via d.w., organs of interest (stomach, duodenum, proximal part of jejunum, liver, and kidneys) were examined in detail by a veterinary pathologist. Representative images of histologic stomach and jejunum sections from control and CAR-treated rats are shown in Fig. 7. No tissue damages or signs of inflammation in any of the investigated organs were observed, and rated histology score values of stomach and small intestine were comparable between groups (control, *n* = 13, 2.31 ± 3.20, range 0–10; CAR, *n* = 14, 1.55 ± 1.37, range 0–4).

## Discussion

Refinement of analgesia in laboratory rats is a measure to improve animal welfare according to the 3R principles but complicated by lack of species-specific data on PK and tolerability for most analgesics commonly in use. Furthermore, analgesia might induce a potential bias on commonly applied behavioral pain indicators. This study was conducted to provide respective evidence-based data for the widely used analgesic CAR in rats of both sexes. In order to assess the feasibility of non-invasive CAR treatment as a means to reduce injection- and handling-associated stress, administration via the d.w. was investigated in addition to s.c. injection.

The study revealed five main findings: (i) A single s.c. injection of 5 mg/kg results in plasma concentrations above the assumed therapeutic threshold for at least 6 h, with C_max_ reached after 3 h and an elimination half-life of 7.06 h. (ii) CAR-medicated d.w. is well accepted with stable fluid intake for at least 5 days. (iii) Despite clear circadian effects on d.w. consumption, oral self-intake of 10 mg/kg/24 h lead to plasma concentrations above the estimated therapeutic level within 24 h and beyond. (iv) Behavioral surrogate pain indicators are not (real-time grimace scale, burrowing) or only mildly influenced (nest-building) by CAR treatment. (v) CAR is well tolerated with only very mild side effects observed for both routes of administration and doses, and prolonged treatment does not lead to gastrointestinal inflammation or ulceration.

CAR is a NSAID commonly used in laboratory rodents to relieve pain and inflammation. It is approved for clinical use in veterinary patients in the EU (various species) and the US (dog) in multiple preparations, but application for pain relieve in rodents is off label. For s.c. injection in rats, dose recommendations range between 2 and 5 mg/kg s.c. with an injection interval of 12–24 h^6,12,15^. Whether meaningful plasma concentrations can be reached and maintained by these doses in rats is not known, however this knowledge is of importance when it comes to evidence-based refinement of analgesic therapy in this species, including the derivation of clinically appropriate application intervals. We decided to evaluate the highest of the currently recommended doses for s.c. injection, and, as no recommendations for treatment via the d. w. are to be found in the relevant literature, to extrapolate the dosing recommended for s.c. treatment for treatment via the d.w.

To the best of our knowledge, this study is the first to investigate PK of CAR after s.c. injection and during d.w. treatment in rats. With 7.06 h, the plasma elimination half-life in rats is slightly shorter than the recently determined 8.52 h for C57BL/6J mice^11^or 10.2 h for CD1 mice^16^. Although in vivo therapeutic plasma levels of CAR in rodents are still unknown to date, current literature often refers to an estimated therapeutic threshold of about 24 µg/ml calculated from in vitro data using canine blood cells (corresponding to a CAR concentration inhibiting 80% of COX-2 activity)^13^. CAR levels were well above this level in most individuals for at least 6 h, indicating that twice daily injection can be generally considered appropriate in rats. Our data suggest sex differences in s.c. PK of CAR in rats, revealing lower plasma concentrations in male individuals up to 12 h after injection. Therefore, for male rats, injection of a slightly higher dose than 5 mg/kg might be necessary to achieve levels above 24 µg/ml in all individuals. Sex differences in PK are also known from human studies^16,17^and currently remain understudied, underlining the importance of including both sexes in preclinical and clinical studies. The non-invasive and stress-free nature of analgesic medication via the d.w. makes this approach favorable from an animal welfare point of view. Of practical relevance, CAR is reported to be stable in d.w. for at least one week^8,18^, which allows for less frequent than daily replacement of the d.w. in daily routine. Our data show that administration of 10 mg/kg/24 h via the d.w. leads to similar plasma concentrations as early after s.c. injection of 5 mg/kg, supporting that this non-invasive treatment route is indeed a promising alternative to achieve stable and relevant plasma concentrations despite the circadian rhythm of fluid intake. Our data show that obtaining plasma levels around an estimated therapeutic threshold might take up to 24 h, suggesting that a pre-treatment period of approximately 1 day prior to surgical procedures may be advisable to ensure that effective plasma or tissue levels are already present at the time of the pain onset. However, as fluid intake can be temporarily reduced in rats after abdominal surgical procedures^19,20^, for example, the actual practical suitability of sole oral self-administration needs to be further substantiated.

Evaluation of potential side effects of analgesics is important as they might put extra burden to the animal but also impact outcome measures of the scientific question. To our knowledge, studies about safety of CAR in rats are not available to date. For tolerability assessment, we therefore closely monitored the rats after single injection and prolonged CAR administration via the d.w. by assessing multiple parameters. In general, rats were in a very good overall condition in this study, and side effects were so mild that they did not become obvious during clinical scoring. Apart from clinical scoring and body weight measurement, a modified Irwin test protocol was performed, as well as hematological and histopathological evaluation was conducted to obtain a comprehensive impression of drug impact when continuously administered for 5 days, a period of time that is often required for the management of moderate to severe post-operative pain. Following s.c. injection, the deviations detected by the Irwin test at the time of the highest plasma levels were very weak, mostly transient, and affected only few parameters. During prolonged treatment via the d.w., the Irwin test revealed only marginal impact on coordination. Interestingly, miosis was found in part of the animals, mainly at time points of highest plasma concentrations. This is surprising and points to a species difference, as NSAIDs in human patients are actually used to prevent miosis^21,22^. However, due to the low degree and transient nature of this autonomous reaction we do not consider it of particular clinical relevance at the given CAR dose.

For both administration routes of CAR, a very mild albeit significant decrease in body temperature relative to baseline (s.c., −0.25 °C; d.w., −0.46°C) was measured in female rats. A reduction in body temperature has been described in female cattle^23^, but is not a typical effect of CAR. Perhaps other mechanisms can underline this phenomenon, since it has been shown that female rats are more sensitive to the antinociceptive effects of NSAIDs^24^. Alternatively, it cannot be ruled out that the body temperature of female rats was influenced by hormonal state and not by CAR treatment. However, the fact that we could not detect any significant differences between the baseline data of female rats speaks against this. Interestingly, in a previous study in mice^11^, a CAR-mediated increase in body temperature was found in both sexes, suggesting species-dependent effects of CAR on this parameter. Therefore, if body temperature is one of the readout parameters measured in research, a potential confounding impact of CAR should be kept in mind.

In the present study, blood count after 5 days of oral treatment revealed increased platelet count as well as increased mean corpuscular hemoglobin (MCH) and mean corpuscular hemoglobin concentration (MCHC) values compared to healthy control rats. Nevertheless, MCH and MCHC values were in physiologic range for both groups according to the manufacturer’s specifications. It is unlikely, but cannot be ruled out, that the repeated blood sampling has led to a certain iron deficiency, which in turn might have caused an increased platelet count even with most other parameters in physiologic range^25^. A systematic review on the impact of NSAIDs on platelet count and function in humans cites a paper reporting an increased platelet count due to aspirin therapy^26^, which would fit with our finding. Altogether, the hematological deviations found in this study were so mild that we do not consider them to be of clinical relevance. Moreover, blood concentration of AST and ALT were not deviant from untreated control rats, indicating that liver function is not impaired by prolonged CAR treatment.

As gastrointestinal side effects might occur after prolonged NSAID treatment^27^, a detailed examination of the gastrointestinal tract was performed in the present study, which did not suggest any adverse effects of prolonged CAR treatment on stomach, duodenum, proximal part of jejunum, liver or kidneys when compared to untreated control rats. This is in line with recent findings in mice, where prolonged s.c. or d.w. CAR treatment regimens were also found to be safe and without adverse histopathological findings^11,28^. Therefore, it can be assumed that the observed increased defecation and soft feces in a few individuals was not caused by CAR treatment. Overall, only minor side effects were observed by the multi-parameter assessment, suggesting a very good tolerability of CAR in the investigated doses in rats.

Grimace scale, burrowing and nest building activity are increasingly applied as non-invasive indicators of pain and distress also in rats^29–33^. Analgesics, however, might inadvertently influence the outcome of these surrogate indicators, which might limit their validity during post-operative pain assessment. On the other hand, awareness of this influence would allow considering this in the assessment. In the present experiments, cage-side (real-time) grimace scale assessment remained unaffected by CAR treatment. As it has been shown that values obtained by real-time scoring can be significantly lower than corresponding scoring of images extracted from video recordings^34^, it cannot be excluded that CAR treatment might still lead to increased score values under different experimental conditions. Burrowing activity remained also unaffected by prolonged CAR treatment. With regard to nest building, male rats showed poor performance overall, irrespective of the experimental phase, questioning the suitability of this parameter for pain assessment in male individuals. In female rats, however, the score values were partially higher after the start of CAR treatment than under baseline conditions. This could have been due to CAR treatment, or an ongoing habituation effect, or both, but might also reflect a certain fluctuation in nest building performance over time as observed during BL2 ^36^. We derive from this that an impact of CAR treatment on burrowing and nesting activity is not to be expected in male rats, but cannot be completely ruled out for female rats when nest building performance is used for pain assessment.

In summary, we found encouraging PK and tolerability profiles for CAR in male and female rats. Only minor effects on Irwin test and hematological parameters were detected, while cage-side surrogate pain indicators and histology remained predominantly unaffected by prolonged treatment. The determined plasma levels indicate analgesic efficacy for at least 6 h already after the first s.c. injection or continuously from 24 h after the start of treatment via d.w. A limitation of our study is that we have not determined the therapeutic dosage regimen of CAR administered via the s.c. and oral routes. However, the present findings may serve as a solid basis for refining CAR-based analgesic protocols for painful conditions in future studies. Provision of CAR-medicated d.w. may contribute to a refined analgesic approach as a monoanalgesic or as part of a multimodal pain therapy, depending on the intended use case or model, as a stress-free method. Despite the stable values observed here for fluid and food intake as well as body weight, we would recommend monitoring these parameters during CAR treatment to ensure animal welfare and intake of the target dose.

## Materials and methods

### Animals and cage set-up

This study was approved by the responsible state authority (Niedersächsisches Landesamt für Verbraucherschutz und Lebensmittelsicherheit) under reference number 33.8–42502-04–21/3640. All methods were performed and reported in accordance with the directive 2010/63/EU of the European parliament and of the council on the protection of animals used for scientific purposes and with the ARRIVE 2.0 guidelines (Animal Research: Reporting In Vivo Experiments; Essential 10). In line with the 3R principles, a longitudinal study design was applied to reduce the number of animals necessary. In-house bred Sprague Dawley rats (RjHan: SD; altogether 55 rats, i.e. 28 male and 27 female rats) were housed in groups of three individuals (experimental animals) or two-three individuals (control animals for histological analysis) per cage (female: type IVS, macrolone, UNO BV, the Netherlands; male: type IV, macrolone Ehret, Germany) on standard bedding material (wood chips from spruce, poplar and aspen trunks, LAB.BED, Thomsen Räucherspäne Räucherholz GmbH & Co. KG, Germany) in a temperature-controlled facility under a 14 h/10 h light/dark cycle (lights on: 6:30 a.m.) with food (altromin 1320 standard diet, Altromin Spezialfutter, Germany) and filtered (particle filter, 5 μm pore size) tap water *ad libitum*. As sex-based differences in PK and behavior have been reported, both sexes were included in this study^29,35,36^. Routine health monitoring according to FELASA recommendations^37^ did not reveal any evidence of infection with common rat pathogens except for *Helicobacter* sp., *Rodentibacter* sp., *Staphylococcus aureus*, *Klebsiella* sp., *Proteus* sp., and apathogenic intestinal flagellates which are known to be present in this housing area. Experimental group size included 21 male and 21 female rats (6 rats per time point (3 male/ 3 female), 7 sampling time points). As control for histological analyses and blood profiles, 7 male and 6 female age-matched rats kept under the same conditions were used. Sample size of *n* = 6 per time point was calculated prior to experiments using G-Power 3.1. No targeted randomization method was used to allocate the rats to experimental and control groups. The rats were randomly allocated to groups of 3 by the animal keepers in the breeding area after weaning from the dam. By establishing the group structure at an early stage, we wanted to prevent the possible occurrence of fighting behavior particularly in the male individuals when moving the rats later on to the experimental area. At start of experiments with age of 20 (male) or 22 (female) weeks, rats weighed 596 ± 47 g (male) and 312 ± 16 g (female). Rats were transferred from the breeding area to the experimental room 3 weeks before start of the experiments. This time was subdivided into one week of adaptation to the new room without particular handling and two weeks of habituation to all handling procedures (Fig. 1a). Precisely, each rat was adapted three times to weighing, manual fixation in a towel, and tail immersion test (for tail immersion test habituation, water with body temperature was used). In their home cages, the rats had continuous access to nesting material (paper pet bedding, ANT Tierhaltungsbedarf, Germany) and wooden gnawing material (1x Aspen Bricks M, 2x Aspen Balls 3 cm, plexx B.V., the Netherlands) as additional enrichment. To minimize potential maintaining-associated confounders, cages were changed weekly always on Monday morning. Home cages were always returned to their original position on the cage shelf. The home cages were all placed on the same side of the animal room and were positioned in such a way that comparable lighting conditions were ensured. All cages were positioned at least 2 m away from the room door.

### General experimental design and blood sampling procedure

An overview of the experimental setup is provided in Fig. 1. Animals were habituated to all handling procedures and interventions (see above) and were trained for burrowing before experiments started. Over 5 days, baseline data (BL1) of food and water consumption, body weight, nest score, Irwin test, rat grimace scale, body temperature, tail immersion test, and burrowing were obtained. This was followed by s.c. injection of CAR and subsequent blood sampling at 1, 2, 3, 6, 12, 24, and 48 h post injection. After 2 weeks of recovery, a second baseline phase (BL2) was performed identical to BL1. Then, CAR was administered via d.w. for 5 days, and blood was collected at 3, 6, 12, 24, 36, 108, and 120 h after start of administration. At all sampling time points, the lateral tail vein of 6 rats (3 male, 3 female) was cannulated and blood (~ 120 µl) was sampled in EDTA tubes (Sarstedt 500µL Microvette^®^^,^ Germany). Immediately after sampling, blood samples were placed on ice, then centrifuged (2500 x g/10 min/4°C), and plasma was stored at −80 °C until further processing. Directly prior to blood sampling, tolerability test (Irwin test) was conducted in the respective 6 rats. At the end of the experiment, rats were euthanized by CO_2_ inhalation, and final blood samples used for full blood count and analysis of electrolytes, glucose, and lactate were gained by cardiac puncture. Further, necropsy of each animal including macroscopic investigation for abnormalities was performed. Stomach, small intestine, liver and kidneys were dissected for histological processing.

### CAR treatment

The CAR dosage for s.c. injection (5 mg/kg, i.e. highest recommended dose) was chosen based on the current GV-SOLAS expert information on pain management for laboratory animals^7^, which is in line with recent suggestions in the upper dose range^6,12^. As the expert information does not recommend a dose for administration via the d.w., this was derived from the highest recommended daily dose for s.c. administration (5 mg/kg every 12 h), resulting in a target dose of 10 mg/kg/24 h via the d.w.. A commercially available CAR solution (Zoetis, Rimadyl^®^ 50 mg/ml, injection solution for dogs and cats) was diluted with 0.9% sterile sodium chloride (B. Braun, Germany) to achieve a 10 ml/kg injection volume for s.c. administration. For d.w. administration, the injection solution (Zoetis, Rimadyl^®^ 50 mg/ml injection solution for cattle) was diluted in filtered tap water. Calculation of CAR concentration was based on mean d.w. consumption/24 h measured over 5 days during BL2 (male rats, 105.43 ± 11.46 ml; female rats, 82.29 ± 8.90 ml) and mean body weight (male rats, 644.71 ± 48.56 g; female rats, 324.33 ± 15.09 g). Administration of CAR-medicated d.w. started on Monday morning (7–8 am) after cage change. Medicated water was freshly prepared every morning in red polyphenylsulfon water bottles (300 ml, Tecniplast, Italy).

### Quantification of CAR in plasma samples and PK analysis

CAR concentration in plasma was quantified by liquid chromatography mass spectrometry (LC-MS/MS). Plasma samples and CAR calibrators (25 µL) were thawed and diluted using 100 µL extraction solvent (acetonitrile/ methanol 1/2) containing 12.5 nM efavirenz (obtained from the NIH AIDS Research and Reference Reagent Program, Division of AIDS, NIAID, NIH) as internal standard (equals a final concentration of 10 nM/sample) in a 1.5 mL reaction tube (SafeSeal^®^, Sarstedt, Germany) for analyte extraction and protein denaturation. Samples were mixed for 30 s using a vortex mixer and frozen overnight at − 20 °C to complete protein precipitation. Then, samples were thawed and centrifuged for 10 min at 20,800 x g/4°C for protein separation. For mass spectrometry analysis, samples were diluted 1:500 using dilution solvent (acetonitrile/methanol/water 2/2/1) containing 10 nM internal standard, and 100 µL were transferred into mass spectrometry vials (Wicom, Heppenheim, Germany) with inserts (Macherey-Nagel, Düren, Germany) for CAR quantification. This involved chromatographic separation on a reversed phase C18-column (ZORBAX Eclipse XDB-C18 1.8 µ, 50 × 4.6 mm, Agilent, Santa Clara, California, USA) connected to a C18 security guard (Phenomenex, Aschaffenburg, Germany) which was kept on 40 °C during the whole analysis. A linear gradient was performed using an HPLC-system consisting of two LC-30AD HPLC pumps, a SIL-30AC temperature controlled autosampler, a DGU-20A5 degasser, a CTO-20AC oven, and a CBM-20 A control unit (Shimadzu, Duisburg, Germany). 10 µL of the sample were injected. Elution started with 80% of solvent A (water + 0.1% formic acid). Within 7 min, the amount of solvent B (methanol 0.1% formic acid) was linearly increased to 95%. This composition was maintained for 3 min followed by a 3 min re-equilibration of the column back to 80/20 (solvent A/solvent B). The total analysis time was 13 min at a flow rate of 0.4 mL/min. The retention time of CAR as well as efavirenz (internal standard) was 7.7 min. Analytes were detected by triple quad mass spectrometry (5500QTRAP; Sciex, Framingham, Massachusetts) in multiple reaction monitoring (MRM) mode. Ionization was achieved using negative electrospray ionization at 650 °C. For CAR, the mass transition m/z 272 → 226 was optimized for quantification, and for efavirenz, the mass transition m/z 314 → 244 was used. Control of HPLC and the mass spectrometer as well as data sampling was performed by Analyst software (version 1.7., Sciex). For quantification, calibration curves were created by plotting peak area ratios of CAR, and the internal standard versus the nominal concentration of seven calibrators containing 8.82 nM – 1000 nM CAR (corresponding to a lower limit of quantification of 0.03 µg/ml and an upper limit of quantification of 0.27 µg/ml) prepared in rat plasma. The calibration curve was calculated using quadratic regression and 1/x weighing.

Pharmacokinetic analysis was performed using the PKanalix application (Version 2024R1, Lixoft, Antony, France). Noncompartmental analysis was applied for determination of plasma elimination half-life of CAR.

### Tolerability assessment

#### Irwin test battery and rat grimace scale

Prior to each blood sampling at 1, 2, and 3 h after s.c. injection and at 3, 6, 12, 24, 36, 108, and 120 h after start of voluntary uptake via d.w. treatment, a modified Irwin test battery (Supplementary Table S1) was performed to detect potential side effects^11,14^. Parameter assessment was subdivided into the categories excitation, coordination, sedation, and autonomous system. Rat grimace scale was scored as an additional parameter of the Irwin test assessment, and visually assessed (without handling of the rats) in the home cage^38^according to an established rat grimace scale score^39^. Immediately after Irwin test procedure, rectal body temperature was measured (PhysioSuite^®^ for mice and rats, Kent Scientific Corporation, USA). At BL2 in male rats, the measurement probe had a spontaneous defect so that no reliable temperature could be measured, and respective data were discarded. Therefore, data from BL1 were used for data analysis. For visualization of Irwin test outcome, a heat map was created showing percentage of changes per investigated time point after treatment (3 male and 3 female rats per time point). For further analysis of the Irwin test outcome, a sum score of each individual rat and test category (excitation, coordination, sedation, and autonomous system) was generated. Total score values were added for a total sum score. Negative score values were handled as positive score values for addition to sum score. Test parameters for which presence or absence were recorded were given 2 points if they deviated from normal. Handling associated vocalization was compared to animal-individual BL and assessed with 2 points when deviant from BL.

#### Food and water intake, and clinical score

Food and water consumption were gravimetrically measured every 24 h per cage during baseline phases and during CAR administration via d.w. For the latter, water bottles were additionally weighed at blood sampling time points (for respective cages), resulting in fluid consumption data for day and night. Clinical score was determined twice a week during baseline phases and daily during CAR administration phases (until day 2 after s.c. injection). Clinical scoring included judgement of activity, general state of health, behavior, body posture and body weight (Supplementary Fig. S5).

#### Behavioral surrogate pain indicators other than grimace scale

A variety of behavioral parameters, such as burrowing and nesting activity and have been suggested as potential surrogate indicators of pain in laboratory rats^29^. They were included in this study to determine potential impact of analgesic treatment on their outcome.

#### Burrowing

Burrowing procedure was performed to investigate motivated goal-directed behavior^29^during CAR administration according to a modified protocol, consisting of habituation, training and experimental phases^32^. For habituation, rats were placed in groups of three (= home cage group) into an empty ‘burrowing cage’ (Makrolon Type IV cage) equipped with two empty burrowing tubes (Polypropylene, length 32 cm, ø 10 cm) for 30 min. Afterwards, rats were moved back to their home cage. For training on four consecutive days, rats were placed in their home cage group into an empty burrowing cage for 10 min. Then, two burrowing tubes filled with 2500 g gravel (Sina BTR Abfüll- und Handels GmbH & Co. KG Germany, natural gravel, ø 2–4 mm) were placed into the cage. After 30 min, rats were moved back to their home cage. Burrowing performance was assessed once per baseline phase and every day (d1, d2, d3, d4) during CAR administration via the d.w. Here, rats were placed alone into the empty burrowing cage for 10 min before each individual was provided with a tube filled with 2500 g gravel for 30 min. Latency to start of burrowing behavior was measured using a stopwatch. After 30 min, the remaining gravel in the tube was weighed and the burrowed amount calculated (2500 g – remaining gravel). For analysis, data recorded at the second baseline (BL2) was used. Burrowing performance was assessed between 9 am and 1 pm.

#### Nest building

Nesting behavior was assessed to detect alterations of intrinsic motivated behavior under drug treatment according to an established nest complexity score^40^. Assessment of nest building behavior was performed using 14 g crinkled paper strips (paper pet bedding, ANT Tierhaltungsbedarf, Germany) per rat, i.e. 42 g per cage. Existing nesting material was taken out and 42 g new nesting material per cage was provided 10 h after start of CAR treatment via d.w., and nest score was recorded for the first time 2 h later. Subsequently, nests were assessed every day in the morning (8–9 am; i.e. 14, 38, 62, 86, 110 h after administration of nesting material) and in the afternoon (4–6 pm; i.e. 24, 48, 72, 96 h after administration of nesting material). Two individual baseline recordings (BL1 & BL2) of nesting evaluation were performed (Fig. 1). For statistical analysis, median and range of BL2 was used.

#### Final blood analysis

After CO_2_ induced asphyxiation, blood from final cardiac puncture was sampled in EDTA tubes (Sarstedt 500µL Microvette^®^, Germany) and immediately stored on ice until analysis. Blood count (scil Vet abc, scil animal care company GmbH, Germany) included white blood cells (WBC), red blood cells (RBC), hemoglobin (HGB), hematocrit (HCT), platelets (PLT), mean corpuscular volume (MCV), mean corpuscular hemoglobin (MCH), mean corpuscular hemoglobin concentration (MCH), and red cell distribution width (RDW). In parallel, additional blood analysis provided status of electrolytes (concentration of K^+^, Na^+^, Ca^2+^, Cl^−^), metabolites (glucose and lactate concentration) and blood (hemoglobin, hematocrit) (ABL815 Flex blood gas analyzer, Radiometer, Denmark). Here, blood from cardiac puncture was sampled in syringes (*safe*PICO Aspirator 1.5 ml, Radiometer, Denmark) and stored at room temperature (< 2 h) until analysis. Additional blood from final cardiac puncture was sampled in EDTA tubes (S-Monovette^®^ 7.5 ml, Sarstedt, Germany), which were immediately placed on ice after sampling, then centrifuged (2500 x g/10 min/4°C), and plasma stored at −80 °C until further processing for AST and ALT measurement. The samples were thawed, and the levels of ALT and AST were analyzed with a Cobas c111 analyzer using manufacturer systems 04718569190/ALTL and 04657543190/ASTL (Roche Diagnostics, Vienna, Austria) for ALT and AST, respectively. The lower detection limit for both ALT and AST was 2 U/L.

#### Necropsy and histology

Directly after cardiac puncture, a routine dissection and visual inspection of the organs in the thoracic and abdominal cavity was performed. Thereafter, stomach, duodenum, jejunum (proximal part), liver and kidneys were harvested. The small intestine was flushed with sodium chloride (0.9% Braun, Germany) and prepared in an improved swiss-roll technique^41^. All organs were placed into formaldehyde solution (4%) for fixation. After at least 3 days of formaldehyde fixation, tissues were trimmed according to the RITA-Guidelines^42–44^, dehydrated (Shandon Hypercenter, XP, Microm GmbH, Germany), and subsequently embedded in paraffin (Histoplast, Thermo Scientific, UK). Sections (2–3 μm thick, microtome Leica RM 2245, Germany) were deparaffinized in xylene and H&E stained according to standard protocols. From each cage of CAR-treated rats, organs of one individual were randomly chosen (https://www.random.org) for histopathological analysis (i.e., 7 male and 7 female rats). For control, organs of 7 male and 6 female age-matched untreated rats ware analyzed. A trained veterinary pathologist performed blinded evaluation (Axioskop 40, Carl Zeiss AG, Germany) and scoring of the sections (stomach, intestine). The kidneys and liver were evaluated for overall pathological findings. The scoring^45^ of the small intestine and stomach considered four, respectively three general criteria: (1) inflammatory cells (2) intestinal architecture, (3) degree of ulceration, if present, and (4) percentage of area involved. The criterion (1) was subdivided into evaluation of severity and the maximal extent of the inflammatory cells regarding the histologic layers of the mucosa and graded from 0 to 4 for each, resulting in a maximum score of 8 for criterion (1). For criterion (2), the mucosal architecture of the intestine was likewise subdivided in evaluation of the epithelial and the mucosal layer and graded from 0 to 4 for each, resulting in a maximum score of 8. The degree of ulceration (criterion (3)) included score values from 0 to 3 and the area involved (criterion (4)) score values from 0 to 4. Score values of all criteria per organ were added up, resulting in an overall maximum score of 23 for the intestine and 15 for the stomach. Images of histological sections were taken on an Axioskop 40 microscope utilizing the software ZEN (Version 3.5 Blue Edition, Carl Zeiss AG, Germany).

#### Statistical analysis

Animal-individual data were recorded for CAR plasma levels, body weight, clinical score, Irwin test parameters, rat grimace scale, body temperature, tail immersion test, hematology, and histology, whereas data for food and water consumption, nesting, and burrowing activity were recorded on cage level. Results are presented as mean and individual data points (plasma concentration-time curves), mean ± standard deviation (CAR intake, burrowing activity, tail withdrawal latency, body temperature, body weight, blood parameters, fluid and food consumption), median and individual data points (sum scores of the Irwin test), or median and range (nest score). Comparisons of two groups were performed by unpaired two-tailed Student’s t-test (blood parameters, burrowing activity BL2 sex comparison) or Wilcoxon matched-pairs signed rank test (body temperature). Multiple comparisons of parametric data were analyzed by one-way ANOVA, followed by Dunnett’s multiple comparisons test (burrowing activity), or Šídák’s multiple comparisons test (food, water, CAR plasma concentrations), or, if not-normally distributed or non-parametric, by Kruskal-Wallis test followed by Dunn’s multiple comparisons test (sum scores Irwin test). Two-way ANOVA followed by Šídák’s multiple comparisons test was performed analyze nesting behavior. GraphPad Prism 9 (Version 9.3.1, GraphPad Software, USA) was used for all statistical analyses. A p-value < 0.05 was considered statistically significant.

Experimental group size for PK profiles oriented on the resource equation method^46,47^. During group allocation, experiment conduction, and data analysis, experimenters (AG, MB) were not blinded. Blinded experimenters performed analysis of CAR plasma concentration using LC-MS/MS (HB), ALT and AST measurement (AK), and histopathological evaluation (SG). If leakage or blockage of water bottles would have been detected, the data from this experimental unit and time point would have been excluded. However, no data were excluded from this study.

## Electronic supplementary material

Below is the link to the electronic supplementary material.

Supplementary Material 1

## Data Availability

Raw data files will be made available from the RepoMed data repository of Hannover Medical School (https://mhh-publikationsserver.gbv.de).
