# Supplementary material for: Favorable pharmacokinetic and tolerability profiles make carprofen an attractive analgesic for subcutaneous injection and oral self-administration in rats
Source: Sci Rep. 2025 Mar 15;15:8932. doi: 10.1038/s41598-025-93336-3 (PMC11909198; doi:10.1038/s41598-025-93336-3)
Supplement: Supplementary file 1 — Supplementary Material 1 [file 41598_2025_93336_MOESM1_ESM.docx]

# **Supplementary Information**

Favorable pharmacokinetic and tolerability profiles make carprofen an attractive analgesic for subcutaneous injection and oral self-administration in rats

Aylina Glasenapp^1^, Jens P. Bankstahl^2^, Heike Bähre^3^, Andrey Kozlov^4^, Silke Glage^1^, Marion Bankstahl^1,5^

^1^Institute for Laboratory Animal Science and Central Animal Facility, Hannover Medical School, Hannover, Germany

^2^Department of Nuclear Medicine, Hannover Medical School, Hannover, Germany

^3^Research Core Unit Metabolomics, Hannover Medical School, Hannover, Germany

^4^Ludwig Boltzmann Institute for Traumatology, Vienna, Austria

^5^Department of Biological Sciences and Pathobiology, Institute of Pharmacology and Toxicology, University of Veterinary Medicine Vienna, Vienna, Austria

**Supplementary Figure 1**

**Supplementary Figure S1.** The sampling time points 1, 2, 3, and 12 h after s.c. injection showed a negative correlation between bodyweight (g) and carprofen plasma concentrations (µg/ml). Pearson correlation was performed for statistical analysis and simple linear regression is plotted as black line in the graphs.

**Supplementary Figure 2**

**a**

**b**

**Supplementary Figure S2**.Calculated carprofen dose intake (mg/kg) within the first 24 h of treatment via drinking water (d.w.). Dose is calculated from individual water intake and concentration per cage and sex **(a)**. In **(b)**, mean carprofen intake (%) during the light and dark phases is shown as a proportion of the total intake within 24 h.

**Supplementary Figure 3**

**a**

**b**

**Supplementary Figure S3.** Consumption/24 h (g) of non-medicated drinking water (d.w.; grey symbols) and carprofen (CAR)-medicated d.w. for 5 consecutive days per sex **(a)**, or food consumption/24 h (g) during baseline measurements with non-medicated d.w. and during CAR treatment over 5 consecutive days per sex **(b)**. Data are shown as mean ± SD (n = 7 cages/sex; n = 3 rats/cage).

**Supplementary Figure 4**

**a**

**Time (h) after start of treatment**

**b**

**Time (h) after start of treatment**

**Supplementary Figure S4**. Daily body weight development of male **(a)** and female **(b)** rats before treatment (baseline 1 and 2, BL1 & BL2), after s.c. injection of carprofen (CAR), and during administration via the drinking water (d.w.). Data points represent values of individual rats (n = 21 per sex), and lines indicate mean ± SD. During baseline phases (BL1 & BL2), rats were weighed 2x/week.

**Supplementary Figure 5**

**a**


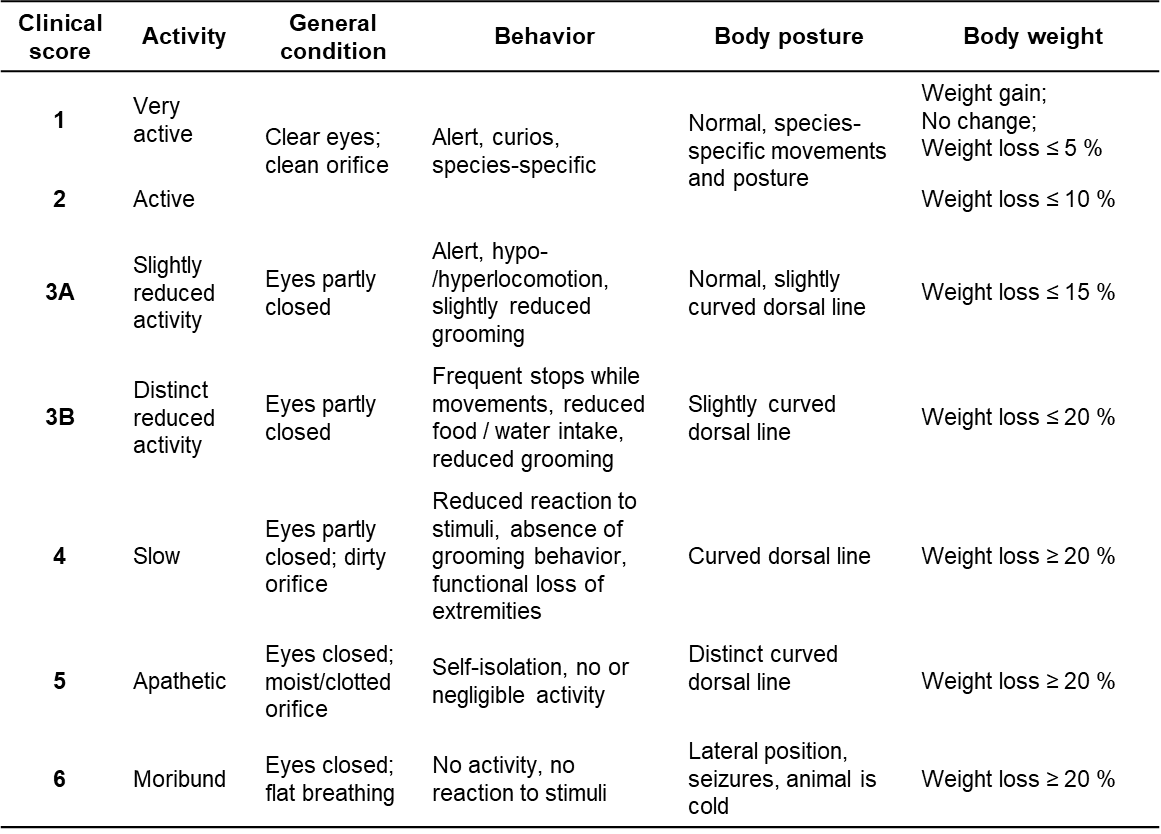


**b**

**Supplementary Figure S5.** Clinical score sheet as applied for daily assessment after subcutaneous (s.c.) injection of carprofen and during drinking water (d.w.) treatment **(a)**. Rats (n = 21 per sex) did not exhibit increased clinical score values at any time point **(b)**.

**Supplementary Table 1**

| **Irwin test parameters (score values)** | **Excitation** | **Coordination** | **Sedation** | **Autonomic** |
| --- | --- | --- | --- | --- |
|  | Max. score per category | | | |
| Irritability to touch (-1; **0**; 1; 2 ; 3) | 3 |  | 1 |  |
| Excitation (**0**; +) | 2 |  |  |  |
| Auditory reflex (fear/startle) (- ; **0**; +) | 2 |  | 2 |  |
| Increased/decreased locomotion (- ; **0**; +) | 2 |  | 2 |  |
| Eyelid reflex associated vocalization (**0**; +) | 2 |  |  |  |
| Pinna reflex associated vocalization (**0**; +) | 2 |  |  |  |
| Stereotypy (2 points per behavior) |  |  |  |  |
| Straub tail (**0**; 1; 2 ; 3) | 3 |  |  |  |
| Jumping (**0**; +) | 2 |  |  |  |
| Exophthalmos (**0**; +) | 2 |  |  |  |
| Increased/decreased respiration rate  (**0**; 1; 2 ; 3; 4) |  |  |  | 4 |
| Tremor (**0**; 1; 2 ; 3) | 3 |  |  |  |
| Myoclonus/Convulsion (- ; +) | 2 |  |  |  |
| Increased/decreased vocalization (- ;+) | 2 |  | 2 |  |
| Abnormal gait (ataxia) (**0**; 1; 2; 3; 4) |  | 4 |  |  |
| Visual placement (-3; -2; -1; **0**) |  | 3 |  |  |
| Grip strength (- ; **0**; +) |  | 2 |  |  |
| Sedation (**0**; +) |  |  | 2 |  |
| Explorative behavior (- ; **0**; +) | 2 |  | 2 |  |
| Ptosis (**0**; 1; 2 ; 3; 4) |  |  | 4 |  |
| Abdominal muscle tone (-2; -1; **0**; +1; +2) | 2 |  | 2 |  |
| Hind limb muscle tone (-2; -1; **0**; 1; 2; 3) | 3 |  | 2 |  |
| Corneal reflex (-2; -1; **0**; +1) |  |  |  | 2 |
| Pinna reflex (-2; -1; **0**; +1) |  |  |  | 2 |
| Body posture (- ; **0**; +) | 2 |  | 2 |  |
| Urination (**0**; +) |  |  |  | 2 |
| Defecation (**0**; +) |  |  |  | 2 |
| Mydriasis (- ; **0**; +) |  |  |  | 2 |
| Miosis (- ; **0**; +) |  |  |  | 2 |
| Lacrimation (**0**; +) |  |  |  | 2 |
| Salivation (**0**; 1; 2) |  |  |  | 2 |
| Diarrhea (**0**; +) |  |  |  | 2 |
| Piloerection (**0**; 1; 2 ; 3; 4) |  |  |  | 4 |
| **Max. sum score** | **36** | **9** | **21** | **26** |

**Supplementary Table S2**: Irwin test parameters, score values for each individual parameter, and maximum possible score per category. Score values in **bold** are considered physiologic.
